# The multifaceted roles of cohesin in cancer

**DOI:** 10.1186/s13046-022-02321-5

**Published:** 2022-03-14

**Authors:** Maddalena Di Nardo, Maria M. Pallotta, Antonio Musio

**Affiliations:** Institute for Biomedical Technologies (ITB), National Research Council (CNR), Via Moruzzi, 1 56124, Pisa, Italy

**Keywords:** Cohesin, Topologically associated domains, Gene expression regulation, Cancer, Chromosome aneuploidy, DNA repair, Replication stress, Genome instability

## Abstract

The cohesin complex controls faithful chromosome segregation by pairing sister chromatids after DNA replication until mitosis. In addition, it is crucial for hierarchal three-dimensional organization of the genome, transcription regulation and maintaining DNA integrity. The core complex subunits SMC1A, SMC3, STAG1/2, and RAD21 as well as its modulators, have been found to be recurrently mutated in human cancers. The mechanisms by which cohesin mutations trigger cancer development and disease progression are still poorly understood. Since cohesin is involved in a range of chromosome-related processes, the outcome of cohesin mutations in cancer is complex. Herein, we discuss recent discoveries regarding cohesin that provide new insight into its role in tumorigenesis.

## Background

Cohesin belongs to the family of SMC (Structural Maintenance of Chromosomes) complexes that organize chromosomal DNA topology in all living organisms, from bacteria to eukaryotes.

**Fig. 1:**
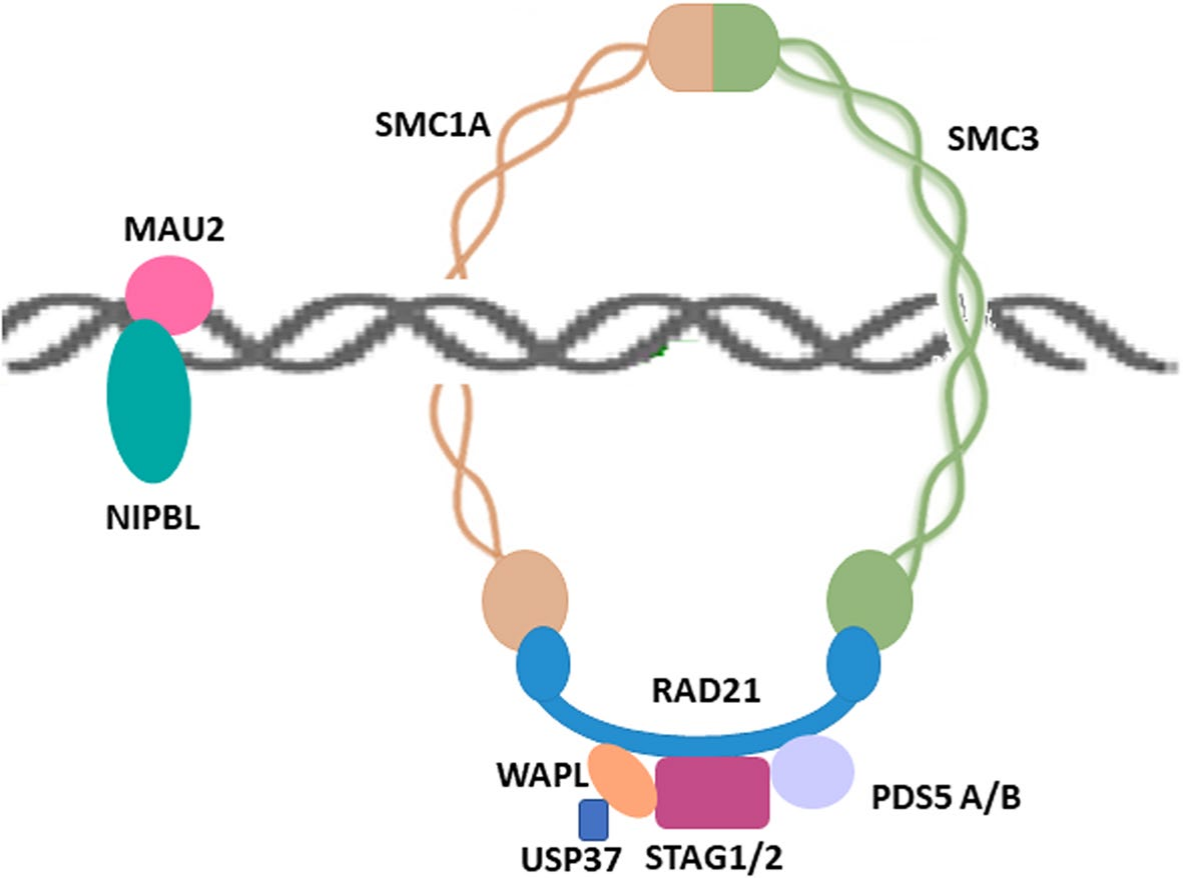
Structure of the cohesin complex. The cohesin ring is composed of SMC1A, SMC3, RAD21, and STAG1/2. SMC proteins are long polypeptides that fold back on themselves to form a coiled-coil domain with a hinge domain at one end and an ATPase domain at the other. SMC1A and SMC3 form a V-shaped structure by interaction of their hinge domains. The N- and C-terminus of RAD21 interact with SMC3 and SMC1A respectively. The STAG1/2 subunit interacts with RAD21.The NIPBL/MAU2 dimer loads cohesin onto DNA, whereas WAPL/PDS5 release cohesin from chromosomes

The core of the cohesin complex is composed of four subunits, SMC1A, SMC3, RAD21/Scc1, and STAG (Stromal antigen)/SA/Scc3, with a ring-shaped structure. SMC1A and SMC3 are characterized by a globular flexible hinge domain bordered by two coiled-coil domains, which fold back on themselves at the hinge, forming a long antiparallel alpha-helical coiled-coil arm that conveys the C- and N-termini together. This latter holds the Walker A box, which binds ATP, whereas the C-terminal contains the Walker B, binding to DNA. SMC1A and SMC3 dimerize at the hinge domains on one side, forming a V-shaped structure through hydrophobic interactions, and RAD21 closes the ring by connecting the SMC1A and SMC3 head domains on the other side. The fourth subunit, STAG1 or STAG2, binds to cohesin by contacting RAD21 and SMC subunits. STAG1/2 are essential for the association of cohesin with DNA (Fig. 1, Table 1) [1].

**Table 1:** Classification and function of cohesin subunits

Gene	Saccharomyces cerevisiae	Drosophila melanogaster	Function
ESCO1/2	Eco1/Ctf7	Deco	Acetyltransferases, establishment of cohesion
HDAC8			SMC3 deacetylase
NIPBL	Scc2	Nipped-B	Cohesin loading
PDS5A	Pds5A	Pds5	Cohesin dissociation
PDS5B			Cohesin dissociation
RAD21	Mcd1/Scc11	Rad21	Core cohesin subunit
STAG1	Irr1	Sa	Cohesin subunit
STAG2	Scc3	Sa2/Stromalin	Cohesin subunit
MAU2	Scc4	Mau2	Cohesin loading
SECURIN (PTTG1)	Pds1	Pim	Separase inibitor
SEPARASE (ESPL1)	Esp1	Sse	Cohesin cleavage
SMC1A	Smc1	Smc1	Core cohesin subunit
SMC3	Smc3	Cap	Core cohesin subunit
USP37			Cohesin dissociation
WAPL	Rad61/Wpl1	Wapl	Cohesin dissociation

Cohesin is highly concentrated at the centromeric regions, while it has a frequency of only 10 kbp in yeast and up to 100 kbp in the higher organisms, along the chromosome arms [2, 3]. Cohesin activity during the cell cycle is regulated by interaction with several regulatory factors (Table 1). During the G1 phase in yeast or at the end of telophase of the previous cell cycle in mammalian cells, the cohesin complex is loaded onto chromatin in cooperation with the activity of the auxiliary factors NIPBL (Nipped-B like)-MAU2. The interaction of cohesin with sister chromatids is established by ESCO 1/2 (Establishment of cohesion 1 homolog 1/2) Eco1/Ctf7 that acetylates the SMC3 subunit during the S-phase. PDS5A/B (Precocious Dissociation of Sisters 5) are also involved in this process. In fact, they interact with cohesin for its establishment and maintenance [4–7].

Cohesin dissociation from chromatin requires the activity of WAPL (Wings Apart-like Protein 1) which interacts directly with RAD21 and STAG1/2 [8, 9]. Recently, it has been shown that WAPL is deubiquitinated by USP37 (Ubiquitin-specific peptidase 37) during mitosis, thereby regulating chromosomal segregation, cohesion and mitotic progression [10, 11]. Finally, once chromosomes are correctly bioriented on the mitotic spindle at anaphase, cohesin is completely removed from chromosomes by the endopeptidase ESPL1 (Extra spindle pole bodies-like 1)/SEPARASE protein that cleaves RAD21 [12, 13]. This cleavage permits opening of the cohesin ring, causing it to dissociate from chromosomes and causing sister chromatid separation [12]. SEPARASE is activated by the proteolysis of its inhibitory partner PTTG1 (Pituitary tumor transforming gene 1) /SECURIN and the simultaneous degradation of CDK1’s subunit cyclin B [14, 15].

The cohesion of sister chromatids and resultant correct chromosome segregation are the best-known functions of cohesin. However, over the last few years increasing experimental evidence has brought to light its key roles in regulating gene expression by mediating functional connections between promoters and their distal enhancers [16, 17], in promoting DNA repair by homologous recombination and non-homologous end joining [18–20], in controlling fork replication stability [21, 22] and facilitating the recruitment of proteins involved in the activation of the intra-S and G2/M checkpoints [23, 24].

Germline pathogenic variants in cohesin core genes and associated factors are responsible for a class of human rare diseases collectively called cohesinopathies or DTRs (disorders of transcriptional regulation) [25]. Variants in *NIPBL*, *SMC1A*, *SMC3*, *HDAC8*, *RAD21*, *BRD4, ANKRD11, ESCO2* and *AFF4* genes are indeed associated with Cornelia de Lange syndrome, Roberts syndrome, and CHOPS syndrome (Cognitive, Heart defects, Obesity, Pulmonary and Short stature), the most frequently encountered and investigated diseases linked to cohesin dysfunction [26–34]. Of note, these diseases are characterized by gene expression dysregulation and impairment in DNA repair [35–41]. Somatic variants and gene dysregulation are instead associated with several types of cancer [42–44] including CRC (colorectal carcinoma) [45–48], breast cancer [49, 50], lung carcinoma [51], UBC (urothelial bladder carcinoma) [52–56], Ewing’s sarcoma [57–59], glioblastoma [60, 61], melanoma [62] and myeloid neoplasms [63–66].

The evolving realization that cohesin participates in a growing assortment of chromosome and chromatin-related processes suggests that its contribution to cancer development is complex. In this review, we summarize recent advances in the understanding of cohesin function in cancer pathogenesis.

## Cohesin, topologically associated domains and CCCTC-binding factor

**Fig. 2:**
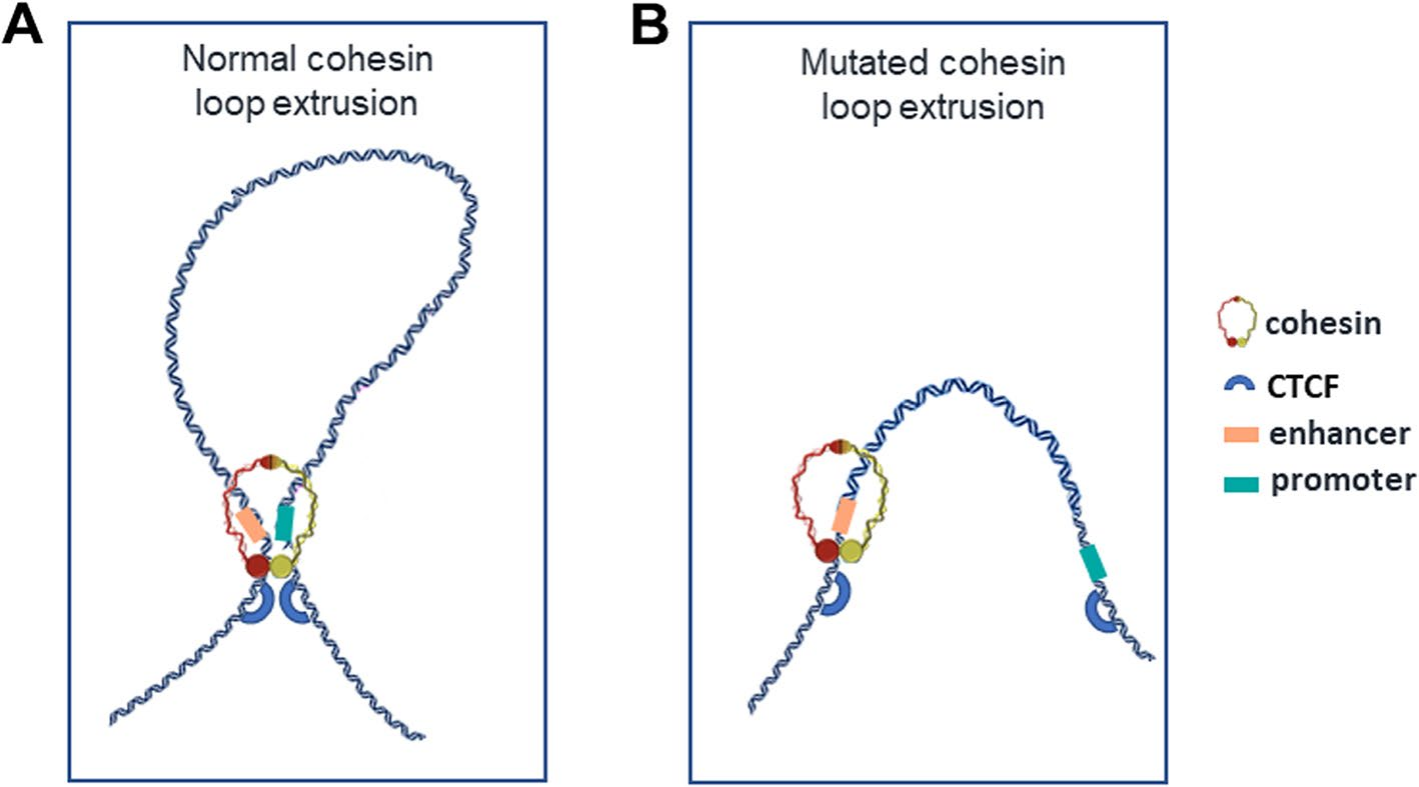
Schematic illustration of the normal and mutated loop-extrusion mechanism. **A** Hypothetical structure of CTCF defined chromatin loop. CTCF stabilizes cohesin in the depicted conformation. **B** Example of abnormal loop formation mechanism

Mammalian genomes are organized at multiple levels. In fact, DNA forms complexes with many proteins at different levels of what is known as the higher order chromatin organization in order to efficiently compact itself. The cohesin is an architectural protein complex involved in gene compartmentalization, enhancer/promoter communication and in organizing the genome into regions called TADs (Topologically Associated Domains). The precise nature and definition of TADs remains a matter of debate. TADs appear to play a double action: to increase the possibilities that regulatory elements meet each other within a single domain, and to segregate physical interactions across boundaries, thus decreasing the chance that detrimental interactions occur [67]. In mammalian cells, TADs range in size from a few 100kbs to 5Mbs in size (with an average of 1MB). The findings that they exhibit a high degree of conservation between cell types and species suggested that TADs represent the fundamental unit of physical organization of the genome [68]. TAD boundaries strongly correlate with replication-timing domains [69] and are enriched for insulator elements such as CTCF (CCCTC-binding factor) [68, 70]. CTCF is an 11-zinc finger DNA-binding protein conserved across most animals, but absent from plants, *C. elegans* and yeast [71]. All interactions mediated by CTCF require the cohesin complex [72–74]. In fact, CTCF directly interacts with the cohesin, and it has been proposed that cohesin extrudes DNA loops until it is arrested by CTCF bound to DNA in a certain orientation or other barrier proteins [75–78]. These loops facilitate the interactions between enhancers and promoters (Fig. 2) [79, 80]. In this process, loop domains prevent enhancers from forming incorrect interactions with targets that are placed in a different loop domain [81, 82]. In the absence of WAPL, PDS5A and PDS5B proteins, cohesin forms extended loops, presumably by passing CTCF sites [74, 83]. In detail, CTCF blocks the cohesin complex by acting as a "boundary" if the 3’ ends of the CTCF binding motifs are oriented towards the interior of the TAD [84]. However, in addition to its function as a translocation barrier, CTCF possesses a distinct loop stabilizing activity, which is realized through direct interaction with RAD21-STAG subunits. In fact, the N-terminal segment of CTCF directly engages the RAD21-STAG subcomplex through the CES (Conserved and Essential Surface) domain [78].

## Cohesin and DNA repair

Genomic integrity is continually threatened by endogenous and exogenous damaging factors such as oxidative damage during metabolism, bases hydrolysis, X-rays, ultraviolet light, and various chemicals. Every day, human cells experience approximately 70,000 DNA lesions, about 75% of them SSBs (Single-Strand Breaks) [85, 86]. SSBs can also be converted to DSBs (Double-Strand Breaks) which, although less much frequent, are highly deleterious. Unrepaired DSBs can generate chromosome translocations, deletions, and insertions, which in turn could lead to genome instability and cancer development. During their evolution cells have acquired highly conserved mechanisms to detect and repair these lesions, thereby restoring genome integrity.

The cohesin complex facilitates the recruitment of proteins involved in cell cycle checkpoints and is also required for DNA damage-induced intra-S phase and G2/M checkpoints in mammalian cells [23]. In fact, cohesin subunits are substrates of ATM (Ataxia Telangiectasia Mutated) and ATR (Ataxia Telangiectasia and Rad3 related) protein kinases activated by specific damaged DNA. ATM phosphorylates SMC1A at Ser957 and Ser966 residues at the intra S-phase checkpoint following irradiation [87, 88]. Instead, ATR phosphorylates SMC1A at Ser957 in response to replication stress [24]. Intriguingly, both human and murine cells carrying mutated or non-phosphorylable SMC1A sites showed decreased cell survival as well as defects in DNA repair [88, 89]. DSBs are repaired by two distinct pathways called HR (Homologous Recombination) and NHEJ (Nonhomologous End-Joining). During HR, the DSB is repaired by exchanges of equivalent regions of DNA between homologous chromosomes, whereas NHEJ reunites the ends without the use of a template. This means that HR-mediated repair is high-fidelity HR, and it is mainly active during the S and G2 phases, whereas NHEJ frequently leaves deletions or insertions at the breakpoint and therefore tends to be error prone.

Cohesin recruitment is fundamental for efficient DSBs repair by HR and this function depends on its ability to mediate cohesion between sister chromatids [19]. Experimental evidence suggests that DSBs allow the establishment of *de novo* sister chromatid cohesion in G2 cells, implicating damage-recruited cohesin in holding the broken chromatid near its undamaged sister template [90, 91]. Moreover, specific recruitment at damaged sites was observed in laser-induced DNA-damage [92]. In human cells, it was recently shown that a DSB unidirectionally blocks cohesin translocation, creating a pattern reminiscent of a TAD boundary. Inside this TAD, cohesin complexes anchored at DSBs extrude chromatin, while ATM phosphorylates chromatin as it passes through the cohesin ring [93]. These findings indicate that genome organization mediated by cohesin is critical for the response to DNA damage.

Instead, NHEJ is active during the cell cycle, and it is the principal pathway during the G1 phase, when there is no immediate close template for homologous repair. The recruitment of DNA–PKcs (DNA-dependent Protein Kinase catalytic subunit) and Ku70/80 to DNA ends triggers the NHEJ cascade, which is followed by enrolment of the XRCC4–ligase IV complex. This process also requires several DNA damage sensors or adaptors, such as ATM, γH2AX, 53BP1, MDC1, RNF168, and the MRE11–RAD50–NBS1 complex. In mammalian cells, the end-joining of the DSEs (Double-Strand DNA ends) is essential in CSR (Class Switch Recombination) and in V(D)J recombination, as well as for repair of DSBs generated by irradiation [94]. It has been hypothesized that cohesin represses the end-joining of distant DSEs specifically in the S/G2 phases while it allows the end-joining of close ends, even in the S/G2 phases [20]. CSR is initiated by recruitment of AID (Activation-Induced cytidine Deaminase) and the subsequent generation of DSBs. As a consequence, AID associates with subunits of cohesin and these breaks activate the DNA damage response and are resolved through the NHEJ pathway [95].

## Cohesin alterations in human cancer

Cancer genome and exome sequencing has revealed that cohesin subunits undergo a wide spectrum of somatic mutations in cancer. According to the COSMIC (Catalogue of Somatic Mutations in Cancer) database (https://cancer.sanger.ac.uk/cosmic) both cohesin core and associated factor genes are involved in cancer (Table 2, as of February 2022). *STAG1* (5%), *NIPBL* (4.9%), *STAG2* (3.4%) and *PDS5B* (3.4%) are the most frequently mutated in cancer. In addition, *STAG2*, *STAG1, SMC1A*, and *RAD21* are also reported in the Cancer Genes Census catalogue (https://cancer.sanger.ac.uk/census) [96], which contains mutations that have been causally implicated in cancer, suggesting that dysfunction of these genes may trigger the tumorigenesis.

**Table 2:** Cohesin core subunits and its modulators in the COSMIC database

Gene	Samples	Mutations	Mutations/Samples (%)	Cancer Genes Census
ESCO1	40,475	618	1.5	No
ESCO2	40,611	369	0.9	No
ESPL1	40,952	961	2.3	No
HDAC8	40,702	910	2.2	No
MAU2	40,484	473	1.2	No
NIPBL	41,039	2019	4.9	No
PDS5A	40,388	1094	2.7	No
PDS5B	41,520	1399	3.4	No
PTTG1	40,832	130	0.3	No
RAD21	63,847	871	1.4	YES
SMC1A	44,193	747	1.7	YES
SMC3	44,590	797	1.8	No
STAG1	41,564	2087	5.0	YES
STAG2	68,769	2321	3.4	YES
USP37	41,001	887	2.1	No
WAPL	40,478	803	2.0	No

In somatic vertebrate cells, two versions of cohesin cohabit, cohesin-STAG1 and cohesin-STAG2, [97]. STAG1 and STAG2 are composed of about 1250 amino acids and share about 75% in homology in their core region while the N- and C-terminal domains are more divergent [98]. Cohesin-STAG2 is more abundant than cohesin-STAG1 in HeLa and *Xenopus* somatic cells; on the contrary *Xenopus* eggs contain more cohesin-STAG2 [99, 100]. The two versions of cohesin complex play different biological functions. In fact, knockout mouse models indicate that *STAG1* plays a pivotal part in telomeric cohesion whereas *STAG2* plays a prominent role in cohesion at chromosome arms or in centromeric regions [101, 102].

*STAG2* is a frequent target of inactivating mutations in human cancers, which are only partially compensated for by its paralogue, *STAG1* [103, 104]. The first evidence of its involvement in tumorigenesis was carried out from focal deletions on the X chromosome observed in glioblastoma [105]. Later, point mutations were identified in UBC [52–55, 106], melanoma [105], myelodysplastic syndrome, acute myeloid leukemia [63, 64] and Ewing's sarcoma [57, 58]. *STAG2* mutations are usually frameshift, nonsense, or splice site mutations leading to absence of proteins [107] though gene deletion and changes in methylation status have also been reported [54, 108, 109]. Approximately 85% of *STAG2* mutations are truncating and often result in loss of expression, indicating *STAG2* as a tumor suppressor gene [104]. The downregulation of *STAG2* in HeLa cells by siRNA has led to the suggestion that impairment of cohesin-STAG2 might be associated with chromosome imbalance [110]. However, cancer cell lines with inactivated *STAG2* were genomically stable though they exhibited decreased cell viability and altered cell cycle. As a consequence, the role of *STAG2* in triggering the aneuploidy associated cancer is still debated [43, 52, 55, 106, 111–113].

According to genomics datasets, 2087 mutations (as of February 2022) have been identified in *STAG1* coding sequences in 41564 tested samples. *STAG1* is frequently mutated in bladder cancer [52, 56], Ewing's sarcoma [59] and myeloid malignancies [64, 65]. About 80% of mutations are missense [114] and two hotspots, c.346G>A and c.419G>A, have been detected. Both are described as pathogenic by using the FATHMM prediction algorithm.

As *STAG2*, *SMC1A* maps in X chromosome in a region which escapes X inactivation. *SMC1A* variants have been detected in brain, blood and bladder cancer [52, 65, 115–117] but it is frequently mutated in CRC [45–47]. CRC is the third most common cancer diagnosed in the population and the second leading cause of death from cancer. CRC progresses through a series of histopathologic and clinical stages ranging from dysplastic crypts to malignant cancers. Most of the *SMC1A* mutations identified in CRC samples are missense [45–47]. The transfection of human primary fibroblasts with vectors carrying some of the *SMC1A* mutations identified in CRC has resulted in chromosome aneuploidy, abnormal anaphases, and micronuclei formation [45] suggesting that *SMC1A* might be responsible for the typical chromosomal instability observed in most cases of CRC. In addition, colorectal tissues acquire extra copies of *SMC1A* gene, and its expression was stronger in carcinoma than normal mucosa and adenoma [46, 48]. The increased expression of SMC1A was positively associated with worse clinico-pathologic variables, including increased tumor, node and metastase (TNM) stages [48].

In addition to CRC, *SMC1A* mutations are associated with other human cancers. Interestingly, *SMC1A* mutations have adverse prognostic relevance in acute myeloid leukemia (AML) resulting in significantly shorter overall survival [118]. Mutations are distributed along the length of the coding sequence but are enriched at several hotspots, preferentially at highly conserved residues within the hinge and ATPase domains [119].

Finally, querying the COSMIC database, 871 of 63,847 (1.4%) cancer samples tested harbored somatic mutations in the *RAD21* coding region. These mutations have been mainly identified in haematological malignancies [120]. Instead, overexpression of *RAD21* was observed in gastric tumors [121], prostate carcinomas [122], CRC [123], and breast cancer [124].

## Effects of cohesin dysfunction in cancer

**Fig. 3:**
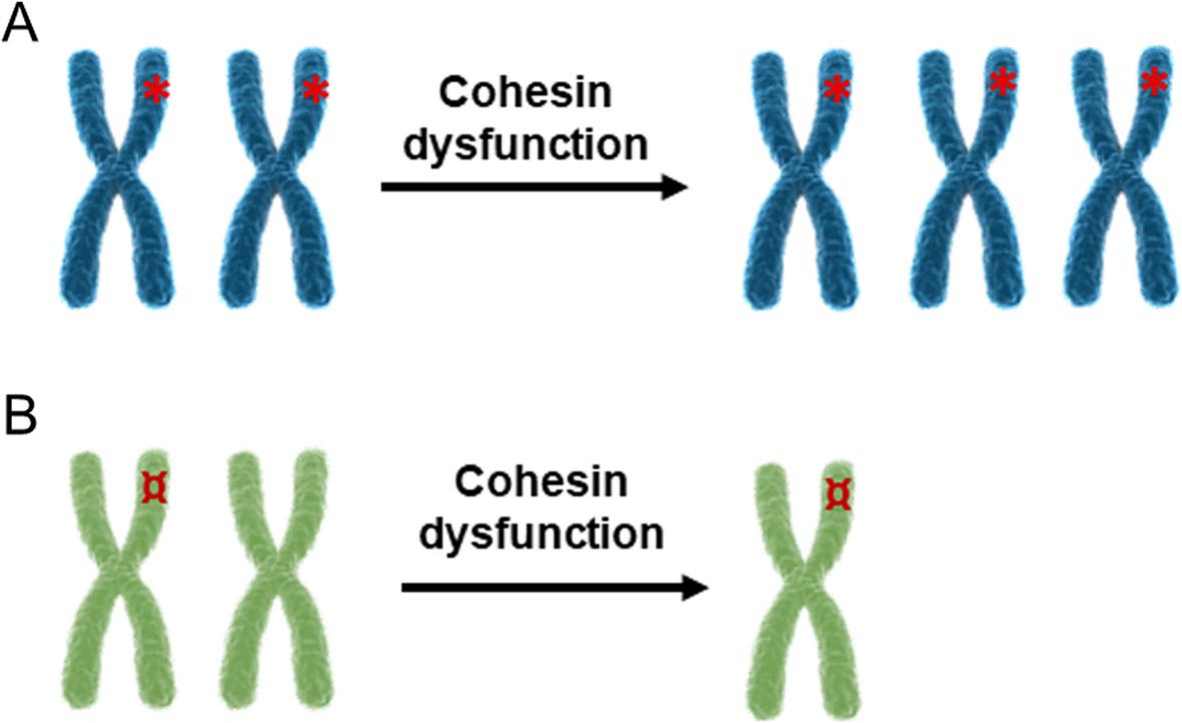
Chromosome imbalance and cancer. **A** Altered segregation of chromosomes harbor a proto-oncogene can lead to gene gain and proto-oncogene over-expression. **B** Knudson’s hypothesis foresees that two hits are required for the inactivation of a tumor suppressor gene. The first hit is an inactivating mutation on the suppressor gene. The second hit is the chromosome loss caused by cohesin dysfunction

Genome instability is a marker of cancer cells. The notion that chromosomal instability may contribute to cancer development was postulated by Boveri more than 100 years ago and later the “aneuploidy first” hypothesis was proposed [125–127]. Mutations and dysregulation of cohesin and cohesin regulatory genes make them powerful driver events that provoke genome instability and cancer progression. The first obvious evidence results from its canonical role. Alterations in cohesin activity lead to impaired chromosome segregation which in turn causes chromosome imbalance, i.e., chromosome gain or loss. The recent query of TCGA (The Cancer Genome Atlas), the largest database of human cancer mutations, showed that half of all driver events are chromosome- and arm-level gains and losses [128]. Each time a chromosome is gained or lost, the dosages of hundreds or thousands of genes are affected. Chromosome segregation impairment can cause trisomy and consequently over-expression of proto-oncogene or transcription factors whose dysregulation play a role in cancer development (Fig. 3). For example, gains of whole chromosome 6 or 6p have been detected in bladder, colorectal, ovarian and hepatocellular carcinomas. It is worth noting that *E2F3* and *ID4* genes, which code for transcription factors, are located on chromosome 6p [129–133].

According to Knudson’s hypothesis, tumor suppressor genes are inactivated by two sequential mutational events or two hits [134]. Cohesin could contribute to one of these hits by chromosome missegregation leading to LOH (loss of heterozygosity) and tumorigenesis (Fig. 3). For instance, in retinoblastoma, one recessive allele of the *RB1* gene may be inherited or result from an early somatic mutation, and the loss of chromosome 13 carrying the *RB1* gene is a frequent second genetic change that leads to LOH of *RB1* [135, 136].

About 85% of CRC is chromosomally unstable, with a worse prognosis. Of note, CRC development is characterized by the gain of several chromosomes containing cohesin genes, such as *HDAC8*, *RAD21*, *SMC1A* and *STAG2* [46]. This finding suggests that cohesin mutations could contribute to generating chromosomal imbalances necessary for a growth advantage and the fully malignant transformation. However, this notion is still under debate. In fact, although *STAG2* is significantly mutated in UBC [137], its alterations occur in the absence of chromosomal instability [52]. Again, no clear association of cohesin mutations and aneuploidy has been reported in myeloid malignancies [107]. Therefore, the role of cohesin dysfunction in cancer development is possibly not only related to cohesion defects and genomic instability, but mutated cohesin may contribute to disease pathology by altering genome structure and gene expression. Aberrant DNA looping could cause misregulation of proto-oncogenes or tumor suppressor genes during tumorigenesis or alter expression of developmental regulators during development and differentiation [81, 138–143].

Cohesin mutations affect the dynamic binding of cohesin onto chromatin and impair the recruitment of Pol II (RNA polymerase II) to both promoter and elongation sites [35, 39]. This data is further supported by the recent findings that cancer-associated mutations identified in *SMC1A*, *STAG1* and *STAG2* genes result in changes to gene expression and genome organization. Mutations interfere with cohesin localization to promoters and enhancers resulting in transcription dysregulation. In addition, mutated cohesin impairs the ability to organize chromatin into loops and the communication between regulatory elements such as enhancers and promoters [144, 145].

*STAG2* LOF (loss of function) occurs in about 20% of Ewing's sarcoma cases [58, 59]. It strongly alters the anchored dynamic loop extrusion process at boundary CTCF sites and dramatically decreases *cis*-promoter-enhancer interactions, which in turn leads to profound changes in the transcriptome. In addition, cells carrying inactivated *STAG2* showed decreased DNA damage signaling and diminished telomere shortening that resulted in delayed senescence. It has been suggested that *STAG2* LOF increases the chance that mutated cells acquire tumor-driving mutations by extending cell life span [146]. This notion is supported by the observation that transcription factors (*MYC*, *NF-κB*) or signaling pathways (epithelial-to-mesenchymal transition, *TGF-β*, and *EGF*) are impacted upon *STAG2* LOF suggesting that these alterations may contribute to tumorigenesis [147].

DNA replication fork progression can be challenged by several factors, such as presence of DNA lesions, inappropriate origin firing, the presence of unresolved DNA secondary structures, deficiency of nucleotide pools available for DNA synthesis, and presence of DNA–RNA hybrid intermediates, leading to transient replication fork progression defects. This replication stress can lead to stalling of DNA polymerases, and prolonged stalling can result in fork breakage due to fork collapse or nucleolytic processing of replication intermediates [148, 149]. The presence of transcriptionally engaged Pol II without productive elongation (promoter-proximal paused Pol II) was first observed for the c-*myc* and c-*fos* genes in mammalian cells [150, 151]. Cohesin has been found to accumulate at stalled forks and its loading depends on chromatin remodeling by the histone acetyltransferase Gcn5 and the H3K4 methyltransferase Set1 [152, 153]. It has been hypothesized that cohesin could facilitate template switching to repair DNA lesions and promote efficient fork restart [153, 154]. The important role of cohesin in resolving replication stress is supported by the observation that its depletion increases Pol II pausing at cohesin binding genes indicating that it regulates its transition to elongation [155]. STAG1 is also involved in this process. In fact, cohesin-STAG1 is implicated in the interactions with the SEC (Super Elongation Complex) involved in mobilization of the paused polymerase [34]. It is interesting to note that alterations in transcriptional control at the level of elongation have been linked to leukemia and multiple myeloma pathogenesis [156, 157].

## Conclusions

In conclusion, cohesin mutations are most commonly found in CRC, bladder cancer, myeloid leukemia, Ewing's sarcoma and glioblastoma. Originally, it was thought that altered cohesin activity was a major cause of aneuploidy in cancer. Instead, increasing evidence indicates that cohesin is a chromatin regulator mediating DNA repair, 3D genome organization, and transcriptional regulation, and changes in chromatin accessibility and transcription are the most striking consequences of cohesin dysfunction in cancer development. A better understanding of how cohesin controls these important biological processes could also lead to the development of novel therapeutic strategies.

## Data Availability

Not applicable.

## Abbreviations

AID: Activation-induced cytidine deaminase
AML: Acute myeloid leukemia
ATM: Ataxia telangiectasia mutated
ATR: Ataxia telangiectasia and Rad3 related
CES: Conserved and essential surface
CHOPS: Cognitive, heart defects, obesity, pulmonary and short stature
COSMIC: Catalogue of somatic mutations in cancer
CRC: Colorectal carcinoma
CSR: Class switch recombination
CTCF: CCCTC-binding factor
DNA–PKcs: DNA-dependent protein kinase catalytic subunit
DSBs: Double-strand breaks
DSEs: Double-strand DNA ends
DTRs: Disorders of transcriptional regulation
ESCO1/2: Establishment of cohesion 1 homolog ½
ESPL1: Extra spindle pole bodies-like 1
HR: Homologous recombination
LOF: Loss of function
LOH: Loss of heterozygosity
NHEJ: Nonhomologous end-joining
NIPBL: Nipped-B like
PDS5: Precocious dissociation of sisters 5
PTTG1: Pituitary tumor transforming gene 1
SEC: Super elongation complex
SMC: Structural Maintenance of Chromosomes
SSBs: Single-strand breaks
STAG: Stromal antigen
TADs: Topologically associated domains
TCGA: The cancer genome atlas
TCGA: The cancer genome atlas
TNM: Tumor-nodes-metastases
UBC: Urothelial bladder cancer
USP37: Ubiquitin-specific peptidase 37
WAPL: Wings apart-like protein 1
